# Prevalence and risk factors for laminitis within the Norwegian pony breed Nordlandshest/Lyngshest

**DOI:** 10.1186/s13028-023-00687-w

**Published:** 2023-06-16

**Authors:** Sigrid Lykkjen, Linda Koldal Stenbakk, Ingrid Hunter Holmøy

**Affiliations:** 1 Department of Companion Animal Clinical Sciences, Faculty of Veterinary Medicine, Equine Section, Norwegian University of Life Sciences, 5003, NO-1432 Ås, Norway; 2 Forus Hesteklinikk, 4031 Stavanger, Norway; 3 Department of Production Animal Clinical Sciences, Faculty of Veterinary Medicine, Norwegian University of Life Sciences, 5003, NO-1432 Ås, Norway

**Keywords:** Age, Breed, Epidemiology, Equine metabolic syndrome, Equine pituitary pars intermedia dysfunction, Insulin dysregulation, Obesity, Prevalence

## Abstract

**Background:**

Laminitis is a systemic condition resulting in debilitating pain and structural changes within the feet, and hence has major welfare implications. Causes include endocrine and systemic inflammatory conditions. Ponies are frequently affected, and observations in the field suggest that occurrence of laminitis is also common in Norwegian breeds. The aim of this study was to estimate the prevalence and risk factors for laminitis within the Norwegian pony breed Nordlandshest/Lyngshest.

**Results:**

The study was a cross-sectional study based on questionnaires sent to members of the Norwegian Nordlandshest/Lyngshest breed association. Questionnaires were received for 504 animals, of which 464 records were eligible and included in analyses. The population comprised 71 stallions, 156 geldings, and 237 mares, with an age between 1 and 40 years (median and interquartile ranges: 12 (6–18) years). The estimated 3-year period prevalence of laminitis was 8.4% (95% confidence interval (CI_95_): 6.0–11.3%), whereas lifetime prevalence was 12.5% (CI_95_: 9.6–15.9%). Mares had a significantly higher period- and lifetime prevalence of laminitis than male horses, and horses 10 years and older had a significantly higher prevalence than younger horses. The lifetime prevalence of laminitis was 3.2% in horses 9 years and younger, whereas from 17.3–20.5% in older horses. Multivariable logistic regression analysis identified age, sex, and regional adiposity as significantly (P < 0.05) associated with the 3-year period outcome of laminitis: Horses older than 9 years had a three-fold increase in the likelihood of having laminitis compared to the younger horses (Odds Ratio (OR)_10–14 years_ = 3.37 (CI_95_ = 1.19–9.50), OR_15-19 years_ = 3.06 (CI_95_ = 1.04–9.05), and OR_>20 years_ = 2.70 (CI_95_ = 0.90–8.02). Mares were more than twice as likely (OR = 2.44 (CI_95_ = 1.17–5.12) to have laminitis compared to male horses, and horses with regional adiposity had increased odds (OR = 2.35 (CI_95_ = 1.15–4.82) of laminitis compared to horses without regional adiposity.

**Conclusions:**

Laminitis appears to be a considerable welfare issue in the Norwegian pony breed Nordlandshest/Lyngshest. The identified risk factors age, sex, and regional adiposity highlight the need for improved owner education and awareness of strategies to reduce laminitis risk.

**Supplementary Information:**

The online version contains supplementary material available at 10.1186/s13028-023-00687-w.

## Background

Laminitis, one of the most debilitating conditions in equids, is now recognized as a clinical syndrome rather than a discrete disease [1, 2]. The condition develops as a sequela to systemic diseases, and it is considered to result from three main categories of disease: (1) sepsis or systemic inflammatory response syndrome, (2) endocrine disease with insulin dysregulation and resultant hyperinsulinemia, or (3) in the supporting limb of a lame horse [1, 2]. Disease processes lead to structural changes and/or failure of the lamellar complex within the foot, causing pain, lameness, and at worst recurrent or chronic conditions that necessitate euthanasia of the animal.

Epidemiological studies have recognized endocrinopathic laminitis (equine metabolic syndrome (EMS) with insulin dysregulation and possibly pituitary pars intermedia dysfunction (PPID)), as the most common form of naturally occurring laminitis [3, 4], and show that ponies and smaller, cold-blooded horse breeds have an increased risk of developing it [3–10]. A systematic review of English language publications in 2011, estimated frequencies of laminitis in worldwide equine populations to range from 1.5% to 34% [11], whereas Knowles et al. [12] recently reported an incidence of 4.8 laminitis cases/100 pony-years in a cohort of nonlaminitic ponies in the south-east of England [12]. In Denmark, it has been shown that cold-blooded breeds, such as Shetland ponies, Dartmoor ponies, Icelandic horses, and Norwegian Fjord horses, have an 18-fold increase in the likelihood of developing laminitis compared to other breeds [6].

Robust frequency data are essential to highlight the importance of a disease, quantify its welfare impact, and allow assessment of preventive interventions. Being able to identify animals at increased risk as well as potential risk factors are obvious key elements to reduce the incidence of laminitis. Observations in clinical practice suggest that the prevalence in native Norwegian breeds is high; however, no epidemiological studies have been conducted. The main objective of the present study was therefore to estimate the prevalence of laminitis within the Norwegian pony breed Nordlandshest/Lyngshest. An additional objective was to describe and categorize breed demographics, dietary and management practices used by owners, body condition scores, as well as laminitis symptoms and treatment characteristics. Based on previous studies that have shown that pony breeds are more at risk of laminitis, it was hypothesized that the frequency of laminitis would be comparable to that of ponies in other countries. Additionally, risk factors for the development of laminitis were proposed to include the presence of endocrine disease and insulin dysregulation, obesity, high energy feeding regimes, access to pasture and sparse exercise regimens.

## Methods

### Study population

The study was a cross-sectional study of the Norwegian pony breed Nordlandshest/Lyngshest and was based on an electronic questionnaire (Additional file 1) distributed to members of the Norwegian breed association. Horses were included if they had lived at least one day in the 3-year study period (2017–2019), including 1 January 2017 to 31 December 2019.

### Questionnaire design and distribution

The questionnaire (Additional file 1) was distributed at the Norwegian breed association website and Facebook-page. In addition, an email and two reminders were sent to all members (*n* = 444) of the association, encouraging them to participate in the study. The members were informed that the aims of the survey were to get information on laminitis frequency and risk factors in Nordlandshest/Lyngshest ponies. Owners were also encouraged to participate in the study if their horse had not had laminitis previously. Data was collected from the 2nd to the 19th of March 2020.

Questions asked included the general demographic data, county, age, and sex of the horses. For the 3-year study period (2017–2019), questions included period-characteristics of housing, exercise, farriery, feeding, pasture, body condition scores, regional adiposity, events of laminitis, and other diseases.

Exercise intensity was defined as high intensity (e.g., race training, hiking with a lot of canter/fast trot, jumping/dressage with a lot of canter and a high heart rate), medium intensity (e.g., working in the arena with more walk and trot than canter, or longer hikes in a lower tempo in varying terrain), and low intensity (e.g., hiking in walk in flat terrain with a low heart rate).

Body condition was scored according to Carroll and Huntington [13]. The scores 0–5, denoted body conditions as very poor, poor, moderate, good, fat, and very fat, respectively. Scoring was conducted by the owners based on an illustration given in the questionnaire. Regional adiposity was determined if the owner (based on a written description) confirmed that the horse had fat accumulations in the nuchal ligament region (cresty neck), behind the shoulder (unilateral or bilateral), around the tailhead, in the supraorbital foramen, and/or in the preputial or mammary gland regions. The occurrence of regional adiposity was analysed as a binary trait, with 0 denoting no regional fat deposits and 1 denoting one or more areas with regional adiposity.

Other diseases included allergic dermatitis, colic, enteritis, endocrine diseases, gastric ulcers, unspecified lameness other than laminitis, metritis, pneumonia, and respiratory diseases. It was not specified whether the diseases were diagnosed by a veterinarian.

Laminitis cases were defined as ponies with either a veterinary diagnosed, or a farrier-/owner-recognized laminitis, as reported by the owners during data collection. Acute laminitis was defined as a horse with clinically apparent laminitis (e.g., bounding digital pulses, hoof warmth, hoof tenderness, and lameness) but without displacement of the distal phalanx. Chronic laminitis was defined as cases with weakening of the lamellae and subsequent displacement of the distal phalanx.

For horses that were reported to have laminitis between 2017 and 2019, questions also included the exact characteristics of housing, exercise, farriery, pasture, and body condition scores at the first event of laminitis (within the period), clinical signs, and treatment. Closed questions were predominant, and response categories were as shown in Tables 1, 2, 3, 4, 5, and 6. Multiple answers were possible for some of the questions.

**Table 1:** Breed demographics

County affiliation, age, and sex	n (%)	Prevalence_2017-2019,_ n (%)	Prevalence _Life,_ n (%)
County			
Agder	16 (3.5)	1 (6.3)	
Innlandet	42 (9.1)	3 (7.1)	
Møre and Romsdal	12 (2.6)	3 (25.0)	
Nordland	99 (21.3)	6 (6.1)	
Oslo	7 (1.5)	2 (28.6)	
Rogaland	23 (5.0)	2 (8.7)	
Troms and Finnmark	111 (23.9)	6 (5.4)	
Trøndelag	48 (10.3)	9 (18.8)	
Vestfold and Telemark	16 (3.5)	1 (6.3)	
Vestland	42 (9.1)	1 (2.4)	
Viken	48 (10.3)	5 (10.4)	
Age			
< 9 years	189 (40.7)	6 (3.2)	6 (3.2)
10–14 years	104 (22.4)	14 (13.5)	20 (19.2)
15–19 years	81 (17.5)	10 (12.4)	14 (17.3)
> 20 years	90 (19.4)	9 (10.0)	18 (20.5)
Sex			
Stallion	71 (15.3)	1 (1.4)	2 (2.8)
Gelding	156 (33.6)	10 (6.4)	17 (10.9)
Mare	237 (51.1)	28 (11.8)	39 (16.5)

The table displays breed demographics of 464 Nordlandshest/Lyngshest ponies and the prevalence of laminitis. The prevalence is given as a lifetime prevalence and a three-year prevalence (2017–2019)

**Table 2:** Management practices

Housing, exercise, training ground and farriery	n (%)	Prevalence_2017-2019,_ n (%)
Housing conditions		
Stabled (daily turnout)	240 (51.7)	25 (10.4)
Outdoor, year-round	204 (44.0)	13 (6.4)
Outdoor, some stabled at night	20 (4.3)	1 (5.0)
Exercise, days/week^*^		
0–3 days/week	327 (70.5)	33 (10.1)
4–7 days/week	137 (29.5)	6 (4.4)
Exercise intensity (*n* = 380)		
Low	109 (28.7)	11 (10.1)
Medium	229 (60.3)	20 (8.7)
High	42 (11.1)	1 (2.4)
Training ground (*n* = 382)		
Soft	33 (8.6)	1 (3.0)
Medium	327 (85.6)	29 (8.9)
Hard	22 (5.8)	2 (9.1)
Farriery routines		
Regular shoeing	199 (42.9)	19 (9.6)
Periodical shoeing	82 (17.7)	7 (8.5)
Barefoot	174 (37.5)	13 (7.5)
Trimmed (young horse)	9 (1.9)	0 (0)
Trimming/shoeing intervals		
≤ 8 weeks	308 (66.4)	23 (7.5)
9–12 weeks	126 (27.2)	14 (11.1)
> 12 weeks	28 (6.0)	2 (7.1)
Never	2 (0.4)	0 (0)

The table displays the distribution of 464 Nordlandshest/Lyngshest ponies by management practices during 2017–2019, and the period prevalence of laminitis

**Table 3:** Dietary practices

Feeding and pasture	n (%)	Prevalence of laminitis (2017–2019), n (%)
Roughage (type)		
Hay (H)	96 (20.7)	14 (14.6)
Vacuum-packed grass (VG)	186 (40.1)	12 (6.5)
Grass silage (GS)	32 (6.9)	3 (9.4)
Roughage combination (H/VG/GS)	150 (32.3)	10 (6.7)
Roughage (Digestible Energy)^*^		
H1 (> 5.9 kJ/Kg)	6 (1.3)	2 (33.3)
H2 (5.4–5.9 kJ/Kg)	71 (15.3)	4 (5.6)
H3 (5.0–5.4 kJ/Kg)	134 (28.9)	17 (12.7)
H4 (4.2–5.0 kJ/Kg)	60 (12.9)	2 (3.3)
H5 (< 4.2 kJ/Kg)	5 (1.1)	2 (40.0)
Unknown	188 (40.5)	12 (6.4)
Concentrates		
High energy product	99 (21.3)	5 (5.1)
Low energy product	144 (31.0)	17 (11.8)
High & low energy	55 (11.9)	2 (3.6)
None	166 (35.8)	15 (9.0)
Concentrates, daily supply		
No supply	133 (28.7)	15 (11.3)
≤ 0.5 L	207 (44.6)	17 (8.2)
0.5–1.0 L	83 (17.9)	5 (6.0)
1–2 L	32 (6.9)	1 (3.1)
2–3 L	9 (1.9)	1 (11.1)
Fruits and vegetables		
Yes	107 (23.1)	5 (4.7)
No	357 (76.9)	34 (9.5)
Vitamins		
Yes	315 (67.9)	30 (9.5)
No	149 (32.1)	9 (6.0)
Pasture		
Infields	63 (13.6)	5 (7.9)
Outfields	146 (31.5)	7 (4.8)
Infields and outfields	242 (52.2)	20 (8.3)
None	13 (2.8)	7 (53.9)

The table displays dietary practices for 464 Nordlandshest/Lyngshest ponies during 2017–2019, and the period prevalence of laminitis

^*^Digestible Energy is given as amount of kilojoules per kilogram of dry matter (kJ/kg)

**Table 4:** Body condition, obesity, additional diseases, and corticosteroid treatment

Body score, regional adiposity, diseases, and corticosteroid treatment	n (%)	Prevalence of laminitis (2017–2019), n (%)
Body condition score (BCS)		
0	0 (0)	0 (0)
1	0 (0)	0 (0)
2	9 (2.0)	0 (0)
3	265 (58.4)	16 (6.0)
4	172 (37.9)	18 (10.5)
5	8 (1.8)	5 (62.5)
Missing	10	
Regional adiposity		
Yes	105 (22.6)	18 (17.1)
No	359 (77.4)	21 (5.9)
Additional diseases in 2017–2019		
Yes	148 (31.9)	28 (18.9)
No	316 (68.1)	11 (3.5)
Additional diseases^*^		
Allergic dermatitis (eczema)	8	3 (37.5)
Colic	59	6 (10.2)
Enteritis	8	0 (0)
Equine metabolic syndrome	3	2 (66.7)
Gastric ulcer	2	0 (0)
Lameness	44	13 (29.6)
Metritis	2	1 (50.0)
Other disease	53	15 (28.3)
Pituitary pars intermedia dysfunction	3	1 (33.3)
Pneumonia	7	0 (0)
Respiratory disease	2	0 (0)
No additional disease	316	11 (3.5)
Corticosteroid treatment for lameness		
Intraarticular	7	1 (14.3)
Intravenous	1	0 (0)
Oral (prednisolone)	3	3 (100)
No treatment	34	9 (26.5)
Corticosteroids for eczema		
Yes	3	2 (66.7)
No	5	1 (20)

The table displays the distribution of 464 Nordlandshest/Lyngshest ponies by characteristics of body condition, obesity, additional diseases, corticosteroid treatment, and the period prevalence of laminitis (2017–2019). The distribution does not include missing values

^*^Some horses had several additional diseases, one horse received two different treatments

**Table 5:** Laminitis characteristics

Laminitis characteristics	*n* (%)
Diagnosed with laminitis (2017–2019)	
Diagnosed by veterinary surgeon	31 (79.5)
Recognized by farrier	7 (17.9)
Recognized by owner	1 (2.6)
Season when recognized	
Winter	7 (18.0)
Spring	5 (12.8)
Summer	16 (41.0)
Autumn	10 (25.0)
Unknown	1 (2.6)
Acute or chronic laminitis	
Acute laminitis	30 (76.9)
Chronic laminitis	7 (18.0)
Unknown	2 (5.1)
Housing when recognized	
Infield, lush pasture	11 (28.2)
Outfield, sparse pasture	15 (38.5)
Stabled, paddock at daytime	9 (23.1)
Other	4 (10.3)
Additional disease when recognized	
EMS/PPID	1 (3.2)
Eczema	2 (6.5)
Unspecified	2 (6.5)
None	34 (83.6)
Coincident event when recognized	
Recent hoof care	2 (5.1)
Grain Overload	2 (5.1)
Lush pasture	13 (33.3)
Pregnant	4 (10.2)
Other	8 (20.5)
None	10 (25.6)
Body score when recognized	
0–2	0 (0)
3	14 (35.9)
4	18 (46.2)
5	5 (12.8)
Unknown	2 (5.1)

The table displays laminitis characteristics of 39 Nordlandshest/Lyngshest ponies that were diagnosed during the 3-year period 2017–2019. The characteristics relates to the first event of laminitis in this period

**Table 6:** Treatment characteristics

Treatment characteristics	*n (%)*
Received treatment for laminitis	
Yes	30 (76.9)
No	5 (12.8)
Euthanized	4 (10.2)
Initial treatment (*n* = 30)	
Box rest	22 (73.3)
Soft bedding	21 (70.0)
Styrofoam pads	4 (13.3)
Trimmed toe	18 (60.0)
Analgesic	24 (80.0)
Cryotherapy	11 (36.7)
Response to treatment (*n* = 30)	
Recovered	12 (40.0)
Recovered, but had recurrent episodes of laminitis	10 (33.3)
Did not recover, euthanized	2 (6.7)
Is still receiving treatment	2 (6.7)
Developed chronic laminitis	4 (13.3)
Number of recurrent episodes (*n* = 14)	
1	2 (5.7)
2	5 (14.3)
> 3	3 (8.6)
Chronic laminitis	4 (11.4)
Prolonged treatment^*^ (chronic laminitis, *n* = 4)	
Corrective hoof trimming	4 (100)
Boots	1 (25.0)
Low-sugar diet	3 (75.0)

The table displays the distribution of treatment characteristics for 39 Nordlandshest/lyngshest ponies that were diagnosed with laminitis during the 3-year period 2017–2019. The characteristics relates to the first event of laminitis in this period. Percentage distribution does not include missing values

^*^Some horses received multiple treatments

### Data analyses

Data handling and analyses were performed using the statistical software STATA (IC v.16.-1) [14].

#### Laminitis frequency

The prevalence of laminitis in Nordlandshest/Lyngshest was calculated as a period prevalence (including the last 3 years, 2017–2020) and a lifetime prevalence. A 95% confidence interval (CI_95_) was calculated for both estimates.

#### Descriptive statistics of questionnaire data

For questionnaire data, the number and percentage distribution of responses across categories were calculated. Missing responses were excluded when calculating percentage distributions.

#### Univariable analyses

The occurrence of laminitis was analysed as a binary trait, with 0 denoting healthy, and 1 denoting having had laminitis in the 3-year period 2017–2019. Univariable logistic regression analyses were performed to investigate associations between potential risk factors (age, sex, housing, exercise, farriery, feeding, pasture, body condition scores, regional adiposity, and other diseases) and the outcome (healthy or diagnosed with laminitis in the 3-year period 2017–2019). Age and sex were also analysed for associations with the lifetime prevalence of laminitis. To avoid mixing up laminitis of different suspected causes, the material was reviewed to identify and exclude cases with concurrent systemic disease, non-weight bearing lameness or corticosteroid treatment. The linearity between the outcome and the continuous explanatory variable age was assessed using the command “lintrend”. No linear relationship was identified, age was therefore handled as a categorical variable.

#### Multivariable analyses

A causal diagram (Additional file 2) was created to visualise relationships and guide model building. Variables showing an association with the outcome in univariable analyses were selected using a significance level of P < 0.2 as a criterion for entry into the multivariable model.

The multivariable logistic regression model was then built using a combination of manual forward selection and backward elimination. Variables retained in the model were significant at a P-value of < 0.05 or were considered potential confounders. Potential confounding variables were identified a priori in the causal diagram. They were then tested by running the model with and without the variables in question while changes in estimates were monitored. Changes in the model estimates of > 30% were used when screening for confounding variables. The overall significance of groups of categorical variables, e.g., age, was tested using likelihood-ratio tests. The effect measure was the odds ratio (OR) with CI_95_.

## Results

### Study population

**Fig. 1:**
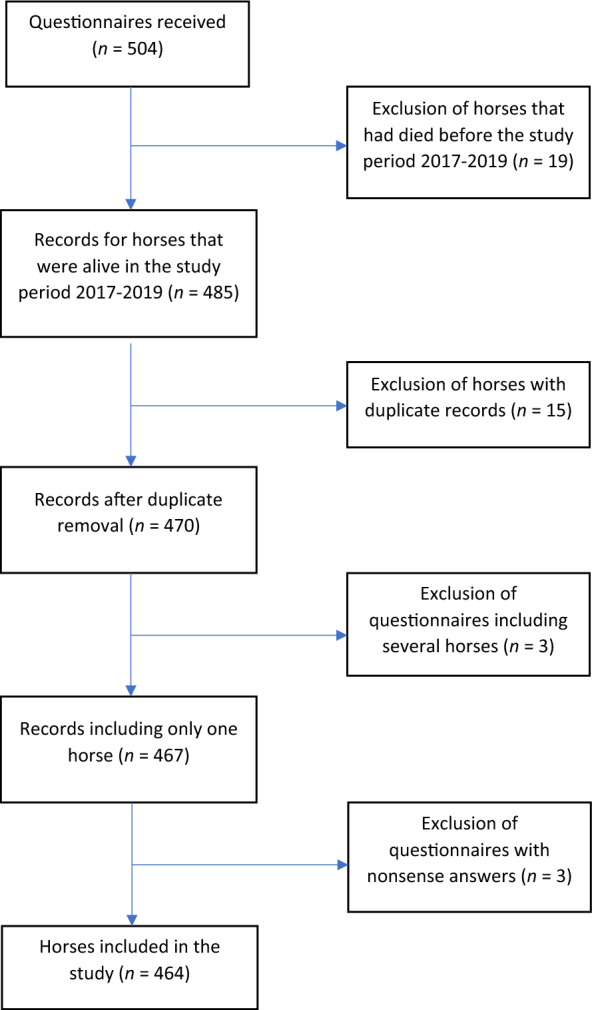
Flow diagram. The diagram summarizes the number of questionnaires received, exclusion criteria and the final number of horses (questionnaire records) included in the analyses

A total of 504 questionnaires were received. After the exclusion of questionnaires that included answers for more than one horse, duplicated questionnaires (different people had answered for the same horse), questionnaires for horses that had died before the study period 2017–2020, and questionnaires with nonsense answers, a total of 464 horses (questionnaire records) remained for further analyses (Fig. 1).

The study-population was distributed across all counties of Norway and constituted 15.4% of horses registered in the national breed association for Nordlandshest/Lyngshest in 2019 (*n* = 3022). The percentages of horses included from each county ranged from 6.3–41.2% (Additional file 3). The population comprised 71 stallions, 156 geldings, and 237 mares (Table 1), with an age between 1 and 40 years (median and interquartile ranges (IQR): 12 (6–18) years).

### Descriptive statistics & univariable analyses

Due to treatment with corticosteroids (concurrent to the laminitis episode), one horse was excluded from both univariable and multivariable analyses. None of the cases had systemic disease or non-weight bearing lameness when laminitis occurred.

#### Laminitis frequency

The study-population had a period prevalence of laminitis of 8.4% (CI_95_: 6.0–11.3), whereas the lifetime prevalence was 12.5% (CI_95_: 9.6–15.9%, Table 1). Mares had a significantly higher period- (P = 0.03) and lifetime prevalence (P = 0.01) of laminitis than male horses, and horses older than 10 years had a significantly higher period- (P = 0.01) and lifetime prevalence (P < 0.001) than younger horses (Table 1, Additional file 4). The lifetime prevalence of laminitis was 3.2% in horses of 9 years and younger, whereas from 17.3–20.5% in older horses (Table 1). The period prevalence of laminitis was 3.2% in horses of 9 years and younger, whereas from 10.0–13.5% in older horses (Table 1).

#### Housing conditions, exercise, and farriery routines

Half the study-population (51.7%) was stabled at night and had daily turnout in a paddock (Table 2). Forty four percent of the horses lived outdoors year-round, whereas 4.2% lived outdoors but were occasionally stabled at night. The period prevalence of laminitis was 10.4% for stabled horses, whereas it was 6.4% and 5.0% for horses living outdoors (Table 2).

Exercise amount and intensity were generally light, as more than 70% of the horses were reported to be ridden only 0–3 days a week (Table 2). The period prevalence of laminitis was 4.4% among frequently exercised horses, and 10.1% in horses that were exercised 0–3 days a week. The difference was on the borderline of significance (P = 0.058, Table 2, Additional file 4).

More than 60% of the horses were regularly or periodically shod, and trimming/shoeing intervals were mostly less or until 8 weeks (Table 2).

#### Feeding and pasture

Vacuum-packed grass and grass silage were given to 47.0% of the horses (Table 3). Hay was given to 20.7% of the horses, whereas 32.3% received a combination of roughages. The amount of digestible energy (kilojoules per kilogramme of dry matter) in roughage was unknown in 40.5% of the cases (Table 3). High-energy (H1-2), medium-energy (H3) and low-energy roughage (H4-5) were given to 16.6%, 28.9%, and 14.0% of the horses, respectively. The period prevalence of laminitis was significantly higher (P = 0.05) among horses that were given only hay (14.6%) compared to horses that received vacuum-packed grass (6.5%, Table 3, Additional file 4).

Concentrates in generally small amounts were given to 64.2% of the horses; furthermore, 23.1% received fruits and vegetables, and 67.9% of the horses received vitamins (Table 3). Most horses were allowed pasture, 52.2% of the horses had a combination of lush infield pasture (high nutrition) and sparse outfield pasture (low nutrition), 31.5% had outfield pasture only, and 13.6% had infield pasture only. Pasture was not allowed for 13 (2.8%) of the horses (Table 3). Among the latter, the period prevalence of laminitis was 53.9%.

#### Characteristics of body condition score (BCS), regional adiposity and additional diseases

Almost 60% of the sample population had a moderate body condition (BCS = 3), whereas almost 40% were scored as fat or very fat (Table 4). None of the horses had a BCS of 0 or 1. Regional adiposity was reported in 22.6% of the horses and included an abnormal distribution of fat with deposits in the crest of the neck or the dorsal aspect of the back and tail head. Although there were few very fat horses (8 horses with BCS = 5), the period prevalence of laminitis was especially high in this group (62.5%). Horses with regional adiposity also had a high frequency of laminitis (Table 4).

Additional diseases during the three-year period 2017–2019 were reported for 148 horses (31.9%, Table 4). Colic, lameness (other than laminitis), and “other diseases” were the most prevalent, noted in 59, 44, and 53 of the horses, respectively. The endocrine disorders equine metabolic syndrome (EMS) and pituitary pars intermedia dysfunction (PPID) were reported in 5 horses only (one horse had both diseases).

#### Laminitis characteristics and treatment of horses that were diagnosed with laminitis

Thirty-nine Norwegian Nordlandshest/Lyngshest ponies were reported to have laminitis during the 3-year period 2017–2019. Most (79.5%) were diagnosed by a veterinary surgeon, while 7 were recognized by a farrier and one by the owner. Laminitis occurrence was most frequent during the summer (41.0%) and autumn (25.0%) seasons (Table 5). Most of the horses experienced acute laminitis (76.9%), whereas 18.0% of the horses developed chronic laminitis following the first episode of laminitis. Housing when diagnosed was distributed on infield pasture (28.2%), outfield pasture (38.5%), stabled (23.1%), and other (10.3%, Table 5). Lush pasture was reported as a coincident event when diagnosed in 33.3% of the cases.

Treatment was given to 30 (76.9%) of the horses and consisted predominantly of box rest (73.3%) with soft bedding (70.0%), trimming of the toe (60.0%), analgesic medication (80.0%), and cryotherapy (36.7%, Table 6). Twenty-two (73.3%) treated horses recovered, 10 (33.3%) had recurrent episodes of laminitis, and four (13.3%) developed chronic laminitis (Table 6). All four horses with chronic laminitis received corrective hoof trimming, and a low-sugar diet was given to 75% of the horses (Table 6).

### Multivariable analyses

Initial multivariable logistic regression analyses included the variables age, sex, exercise, feeding, pasture, BCS, and regional adiposity (P < 0.2 in univariate analyses, Table 1, Additional file 4). Following forward selection and backward elimination, age, sex, and regional adiposity remained in the final model.

Table 7 shows the multivariable adjusted OR for variables that were significantly associated with the occurrence of laminitis. Age groups older than 9 years had tripled odds of laminitis compared to horses aged nine years and younger (Odds Ratio (OR)_10–14 years_ = 3.37 (CI_95_ = 1.19–9.50), OR_15–19 years_ = 3.06 (CI_95_ = 1.04–9.05), and OR _> 20 years_ = 2.70 (CI_95_ = 0.90–8.02). Females were more than twice as likely (OR = 2.44, CI_95_ = 1.17–5.12) to have laminitis compared to male horses. Horses with regional adiposity had increased odds (OR = 2.35, CI_95_ = 1.15–4.82) of laminitis compared to non-obese horses (Table 7).

**Table 7:** Multivariable logistic regression analyses

Predictors	*n*	OR	CI_95_ of OR	P
Age				
< 9 years	189	Baseline		
10–14 years	103	3.37	1.19–9.50	0.02
15–19 years	81	3.06	1.04–9.05	0.04
> 20 years	90	2.70	0.90–8.02	0.07
Gender				
Male	227	Baseline		
Female	236	2.44	1.17–5.12	0.02
Regional adiposity				
No	359	Baseline		
Yes	104	2.35	1.15–4.82	0.02
Intercept		0.017	0.00–0.47	

The table shows the multivariable adjusted odds ratios (OR) for variables that were significantly associated with occurrence of laminitis (n = 463). CI_95_**:** 95% confidence interval**,** P: significance level

## Discussion

The current study estimated the period (2017–2019) and lifetime prevalence of laminitis within the Norwegian pony breed Nordlandshest/Lyngshest of 8.4% and 12.5%, respectively. The variables age, sex, and regional adiposity were identified as significant risk factors for laminitis in the breed. Horses older than 9 years had a three-fold increase in the likelihood of having laminitis, whereas mares and horses with regional adiposity had more than doubled the risk of having laminitis. The findings emphasise that laminitis is a considerable welfare issue in the Norwegian pony breed Nordlandshest/Lyngshest.

This study is the first to estimate the frequency of laminitis in a sample of a native Norwegian pony population. There are no comparable Norwegian studies; however, studies from other countries cover a variety of populations, geographies, situations, and timespans. In specific equine sub-populations, frequencies up to 34% have been reported [15–17], but laminitis frequency estimates from general equine populations are probably more comparable to the present study. A prospective study reported the prevalence and incidence of veterinary-diagnosed active laminitis in the general British horse population as 0.47% and 0.5/100 horse-years at risk (HYAR), respectively [18]. Pollard et al. reported a first episode incidence of 9.6 cases/100 HYAR in a British study based on owner-reported occurrence, and also found a significant difference in laminitis incidence among breeds [5]. For ponies in the south-east of England, an incidence of 4.8 laminitis cases/100 HYAR was recently reported [10]. In Australia, a retrospective study reported that 15% of the animals attending Pony Clubs in Victoria had suffered from at least one episode of laminitis and that ponies were more commonly affected (21.8%, 6.5/100 HYAR) than horses (4.4%, 0.55/100 HYAR) [19]. The present prevalence estimates were higher than the veterinary-diagnosed rates for the general British horse population [18] as well as for horses in Australia [19], but lower than reported in some pony breeds [15–17, 19]. Although study design, data collection methods, and case definitions may vary, frequency differences between horses and ponies are probably best explained by a higher incidence of endocrine disease and -laminitis in ponies [3, 4, 6–8, 10]. For pony populations, breed predisposition and exposure to risk factors for endocrine disease (e.g., increasing age, being a female, obesity, and low activity) may explain differences in laminitis occurrence [3, 5–9]. For example, our population included a large proportion of young animals (24% ≤ 5 years of age), which contributed to an overall low estimate of prevalence; however, for ponies older than 9 years, it was observed that the lifetime prevalence was approaching 20%.

The variables age, sex, and regional adiposity were in multivariable analyses identified as significant risk factors for laminitis in the breed, which corresponds to previously identified risk factors for both laminitis [12] and endocrine diseases [3, 9] in UK ponies and cobs. The increasing risk of laminitis in older horses may be explained by longstanding exposure to risk factors as well as the association between age and endocrine disorders [3, 9]. In the current study, horses older than 9 years had tripled odds of laminitis, but the laminitis risk also appeared to slightly decrease in the older age categories. The reasons for this were not further investigated but may be explained by euthanasia of horses with recurrent laminitis or that owners learned to modify the laminitis risk factors.

Our survey specifically questioned but did not identify any laminitis cases that were related to foaling complications such as retained placenta and metritis. Nevertheless, it was found that mares were at increased risk of laminitis compared to both geldings and stallions. This is consistent with findings in previous studies, which show that mares have an increased risk of developing both endocrine disorders and laminitis [9, 15, 16].

In the present study, ponies with endocrine diseases also presented with a high period prevalence of laminitis (66.7% and 33.3% for EMS and PPID, respectively); however, as only 5 cases were identified (1.1% of the study population), the variables were not verified as risk factors. Equine metabolic syndrome is a recognized collection of risk factors for endocrinopathic laminitis, in which insulin dysregulation represents the defining feature of the condition [20]. In UK native ponies and cobs, the prevalence of EMS was recently estimated to 23.3%, and the risk factors for EMS, including increasing age, being female, obesity, less strenuous exercise, and shorter periods on pasture during the summer, were also identified [9]. In Norway, it is currently less common to carry out tests for insulin dysregulation in ponies, nor is it recognized that most overweight ponies suffer from EMS. However, as our identified risk factors for laminitis also represent risk factors for EMS [9], it is plausible that the actual prevalence of EMS in our population is far higher and perhaps close to the prevalence of regional obesity. Furthermore, our study did not reveal laminitis cases related to concomitant systemic disease or prolonged abnormal weight bearing related to severe lameness, and it is therefore reasonable to believe that EMS with insulin dysregulation was the main cause of laminitis in our study. This highlights the need for improved diagnosis and monitoring of EMS to help identify at-risk ponies, as well as further research to identify risk factors for EMS in the Norwegian breed. An English study recently reported laminitis risk in previously non-laminitic ponies, to be best identified by basal or oral sugar test stimulated serum insulin concentrations [12].

Previous studies have found that the rate of laminitis was higher in horses that were lame or foot sore after previous shoeing/trimming [7, 10] and that shoeing/trimming intervals of > 8 weeks were associated with higher rates of laminitis [10]. We found no effect of farriery routines, trimming, or shoeing intervals on the prevalence of laminitis.

Exercise amount and outdoor housing on the other hand, were in univariate analyses significantly associated with a reduced prevalence of laminitis. Alford *et.al* [21] compared the level of exercise between horses affected by acute laminitis and controls and found that cases were significantly more likely to be in the little or no regular exercise category compared to the moderate or strenuous category. It is known that exercise has beneficial effects on glucose homeostasis, and can enhance insulin sensitivity, and may therefore have a direct role in preventing laminitis through physiological effects and/or by reducing obesity [22, 23]. Outdoor housing encourages activity and counteracts obesity; however, the lower prevalence in this study may also be explained by preventive measures, i.e., less turnout on grass for ponies with previous laminitis.

Previous studies have evaluated pasture- and turnout-related risk factors for laminitis [15]. Wylie *et.al.* found that animals with new access to grass (and no access in the previous 4 weeks), were more than seven times as likely to develop laminitis compared to controls with access for a longer duration or no access at all [7]. Also, a Danish study found that a recent change of grass, and being on high quality grass were significant risk factors for laminitis [6]. In this study, 66.7% of the ponies were at pasture (infield or outfield) at the first laminitis occurrence. Obtained information on feeding and grazing in the three-year period (2017–20), and further identified an increased prevalence of laminitis among horses that were not grazing. This latter finding may be explained by preventive measures, i.e., less or no pasture access for horses previously having had laminitis, which were also observed in the recent studies [9, 12].

The present study obtained information on feeding in the three-year period (2017–20) but lacked information on diet at the first case of illness. Furthermore, more than 40% of the owners did not know what energy level of roughage their horses were receiving. This limits the opportunity to assess whether roughage and concentrates represent risk factors for the condition.

The validity of an internet questionnaire study relies on comprehensive, clear, and unambiguous questions as well as a sufficient return of data. Owner-based questionnaire surveys with voluntary return of data are prone to low responses, as well as response-, non-response-, and recall bias, may limit the generalisability of the findings. This study may have been more appealing to owners with an interest in preventing laminitis due to prior experience with the condition, potentially contributing to an elevated prevalence of laminitis. On the other hand, owners who are not familiar with the clinical signs of laminitis may have contributed to the misclassification bias that lowered the estimate. Here, only one case was diagnosed by the owner, whereas healthy horses were generally classified as such by the owners. Also, not retrieving the exact date for the first laminitis occurrence and deaths within the study-period (2017–2019) represents a limitation of the present study.

## Conclusions

This study provided information on the frequency of laminitis in the Norwegian pony breed Nordlandshest/Lyngshest and indicated that it is a considerable welfare issue in the breed. The identified risk factors age, sex, and regional adiposity highlight the need for increased diagnosis and monitoring of EMS/insulin dysregulation in the breed, improved owner education, and awareness of strategies to reduce laminitis risk.

## Supplementary Information

**Additional file 1:** Questionnaire distributed to members of the Norwegian Nordlandshest/Lyngshest breed association.

**Additional file 2:** Causal diagram for laminitis.

**Additional file 3:** **Distribution across counties**. The table displays the distribution across counties for all horses registered in the Norwegian breed association for Nordlandshest/lyngshestin 2019, and the NL study-population.

**Additional file 4:** **Univariate analyses.** The table displays the number of observations, odds ratio, 95% confidence intervalsand *P*-values for factors associated with outcomewith *P*<0.2, based on univariable testing using logistic regression.

## Data Availability

The datasets used and analysed during the current study are available from the corresponding author on reasonable request.

## Abbreviations

BCS: Body condition scores
EMS: Equine metabolic syndrome
CI_95_: 95% confidence interval
HYAR: Horse-years at risk
OR: Odds ratio
PPID: Pituitary pars intermedia dysfunction
