# Supplementary material for: Prevalence and risk factors for laminitis within the Norwegian pony breed Nordlandshest/Lyngshest
Source: Acta Vet Scand. 2023 Jun 16;65:22. doi: 10.1186/s13028-023-00687-w (PMC10276406; doi:10.1186/s13028-023-00687-w)
Supplement: Supplementary file 1 — Additional file 1: Questionnaire distributed to members of the Norwegian Nordlandshest/Lyngshest breed association. [file 13028_2023_687_MOESM1_ESM.docx]

General questions

1. In which county do you live?

2. What is the name of your horse (official name and pedigree number if available)?

3. What is the gender of your horse?

- Stallion
- Mare
- Gelding

4. In what year was your horse born?

5. How many days a week is your horse being used (when not injured)?

- 0
- 1–3
- 4–7

6. What is the normal activity level when the horse is being used?

- High (high intensity over an extended period of time, e.g. race training, hiking with a lot of canter and/or fast trot, jumping/dressage with a lot of canter work and a high heart rate)
- Medium (e.g. working in the arena with more walk and trot than canter, or longer hikes in a lower tempo in varying terrain)
- Low (e.g. hiking in walk in flat terrain, low heart rate)

7. On what surface area is the horse most often being used?

- Soft surface (e.g. loose sand, deep footing in riding arena)
- Firm surface (e.g. wooded area, gravel roads, firm riding arena, fiber footing in riding arena)
- Hard surface (e.g. asphalt, hard packed gravel)

8. How is your horse’s housing conditions when not outside all day/night for summer pasture?

- Outdoor, year-round
- Stabled at night, paddock/field during the day
- Other

9. If you answered “Other”, could you describe the housing conditions?

10. Is your horse on pasture during the summer months?

- Yes, mainly infields (high nutrition pasture)
- Yes, mainly outfields (low nutrition in varied terrain)
- Yes, infields and outfields
- No

11. What type of roughage does your horse eat?

Choose all relevant alternatives.

- Dry hay
- Vacuum-packed grass
- Grass silage
- Concentrate substitution for roughage

12. How much roughage do you feed your horse?

13. What types of concentrates does your horse eat?

- Low energy (e.g. Champion Betfiber or other concentrates with low sugar/starch content)
- High energy (e.g. Champion Gull, Champion Komplett)
- Fruits and vegetables (e.g. carrots and apples)
- Vitamins and minerals
- None

14. Do you have an nutritional analysis of the roughage you feed your horse?

- Yes
- No

15. What quality is the roughage you feed your horse?

- H1
- H2
- H3
- H4
- H5
- Don’t know

16. Does your horse wear horseshoes?

- Yes
- No
- Sometimes
- Other

17. How often do you trim/shoe your horse?

- Every 8 weeks or more often
- Every 9 to 12 weeks
- Less often than 12 weeks
- Never

18. What body condition score (BCS) is your horse?

- 0
- 1
- 2
- 3
- 4
- 5
- Has changed a lot in the relevant time period (more than 2 scores, e.g. from 2 to 4)


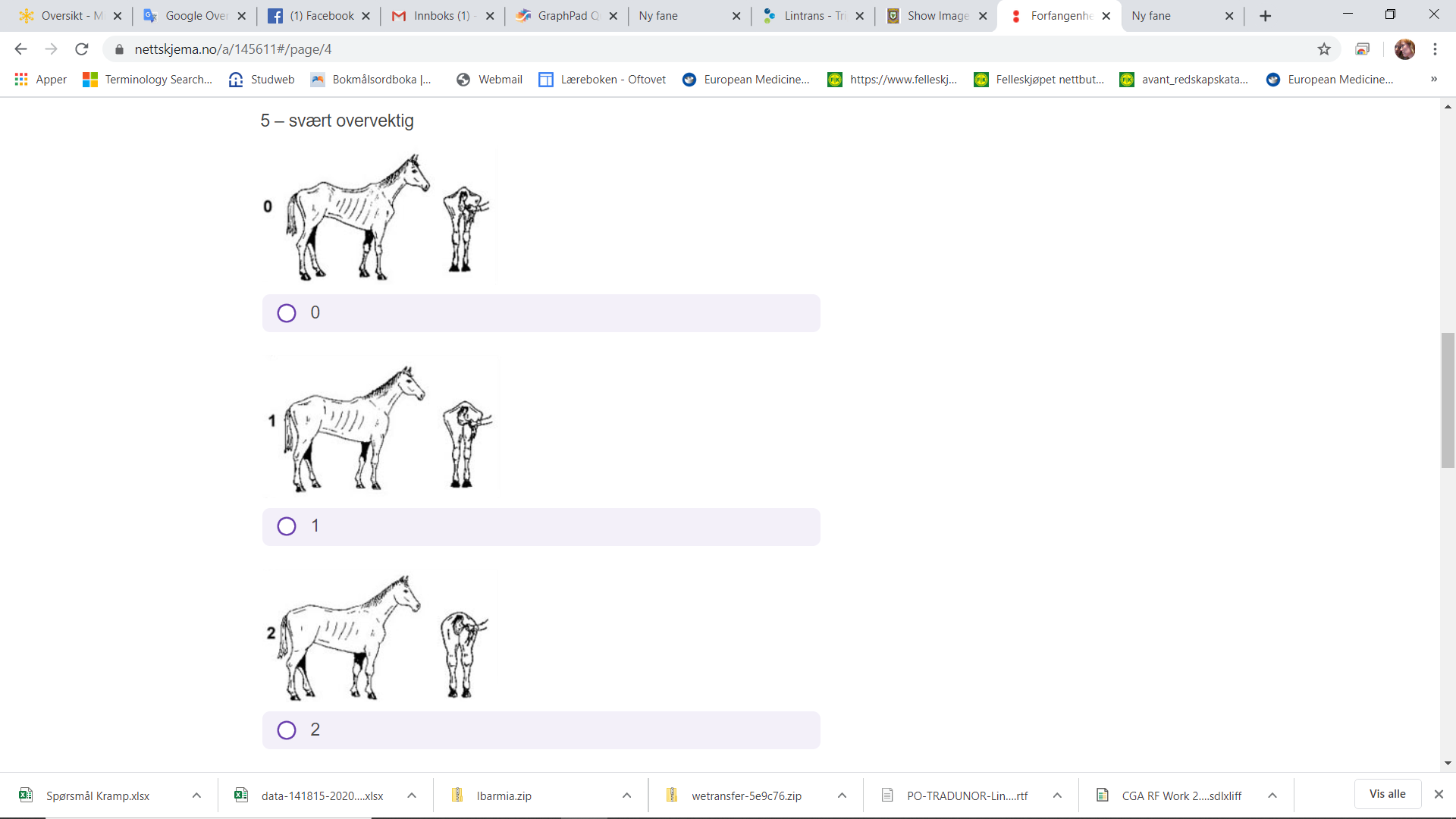


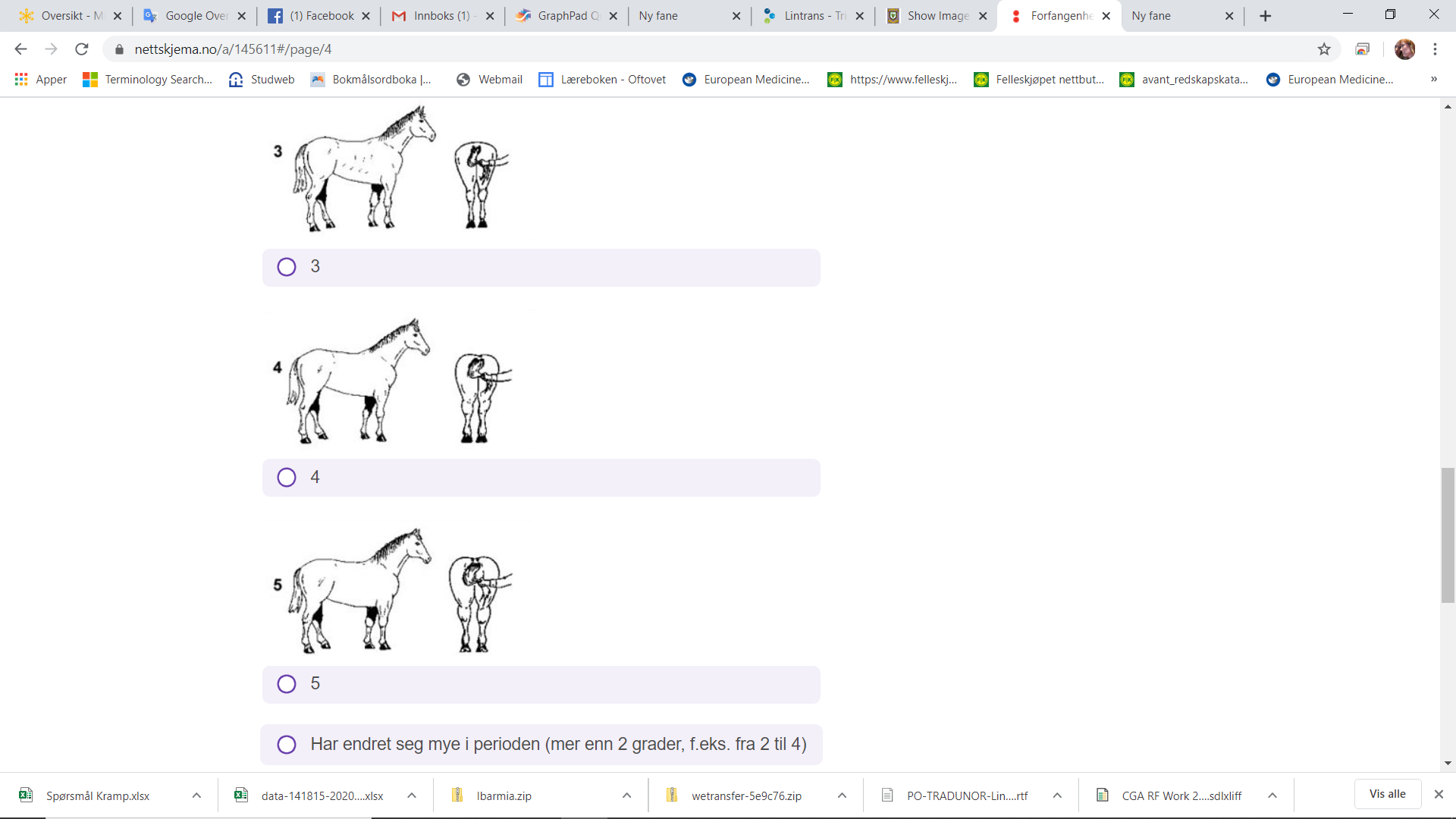


19. Does your horse have any of the following characteristics?

- Heart shaped hindquarters
- Fat deposit on the neck
- Fat deposit behind the shoulder
- Fat deposit over the eye socket
- Swollen around the penis or udder
- None of these

20. Have your horse been sick / been diagnosed with a disease the last three years?

- Yes
- No

21. Which illness/injury did your horse have?

Choose all relevant, apart from laminitis.

- Equine metabolic syndrome
- Cushing/PPID
- Hyperlipidemia/lipidemia
- Lameness
- Retained placenta / metritis
- Airway infection / pneumonia
- Allergic respiratory disorder
- Different respiratory disorder
- Mastitis
- Sepsis
- Colic
- Stomach ulcer
- Intestinal inflammation / colitis
- Summer eczema / allergic skin disorder
- Other illness/injury

22. Has your horse been treated with corticosteroids in relation with lameness / joint disease?

- Yes
- No

23. Which type of corticosteroid treatment did your horse receive?

- Joint injection
- Intravenous injection
- Oral tablets/powder

24. If known, write down which medication was given and for how many days.

25. Has your horse been treated with corticosteroids in relation with allergic respiratory disorder?

- Yes
- No

26. Which type of corticosteroid treatment did your horse receive?

- Joint injection
- Intravenous injection
- Oral tablets/powder

27. If known, write down which medication was given and for how many days.

28. Has your horse been treated with corticosteroids in relation with summer eczema / allergic skin disorder?

- Yes
- No

29. Which type of corticosteroid treatment did your horse receive?

- Joint injection
- Intravenous injection
- Oral tablets/powder

30. If known, write down which medication was given and for how many days.

Questions regarding laminitis

31. Has your horse suffered from laminitis (during its lifetime)?

- Yes
- No
- Don’t know

32. Has your horse been diagnosed with laminitis during the last three years?

- Yes, by a vet
- Yes, by a farrier / barefoot trimmer
- Yes, by someone else than a vet / farrier / barefoot trimmer
- No

33. When during the year was the horse diagnosed with laminitis for the first time?

- Spring
- Summer
- Autumn
- Winter
- Can’t recall / don’t know

34. Was the horse diagnosed with acute or chronic laminitis?

Acute laminitis is an inflammation in the sensitive lamellas of the hoof. Clinical signs can be heat in the hoof, increased digital pulse, tenderness, lameness and sometimes fever and reduced general condition.

With chronic laminitis the connection between the hoof capsule and the coffin bone is destroyed. The coffin bone can rotate or sink within the hoof capsule. Classical clinical signs of chronic laminitis are a change in the hoof growth with a long toe, flat sole, broad white line and diverging lines on the hoof capsule.

- Acute
- Chronic
- Don’t know

35. How were the housing conditions of the horse when it was diagnosed with laminitis the first time?

- Stabled at night, paddock/field during the day
- Infield pasture (high nutrition)
- Outfield pasture (low nutrition)
- Other

36. Was your horse suffering from an illness/injury when it was first diagnosed with laminitis?

- Yes
- No

37. Which illness/injury was your horse suffering from when it was first diagnosed with laminitis?

- Equine metabolic syndrome
- Cushing/PPID
- Hyperlipidemia/lipidemia
- Lameness in another leg
- Retained placenta / metritis
- Airway infection / pneumonia
- Allergic respiratory disorder
- Different respiratory disorder
- Mastitis
- Sepsis
- Colic
- Stomach ulcer
- Intestinal inflammation / colitis
- Summer eczema / allergic skin disorder
- Other illness/injury

38. Had your horse recently been treated with corticosteroids when it was first diagnosed with laminitis?

- Yes
- No

39. If known, write down which medication was given and for how many days.

40. Was one of the following true for your horse when it was first diagnosed with laminitis?

Choose all relevant alternatives.

- Recently trimmed hooves
- Horse had eaten too much concentrates
- Recently let out on lush pasture
- Pregnant
- Other
- No

41. What body condition score (BCS) was your horse when it was first diagnosed with laminitis?


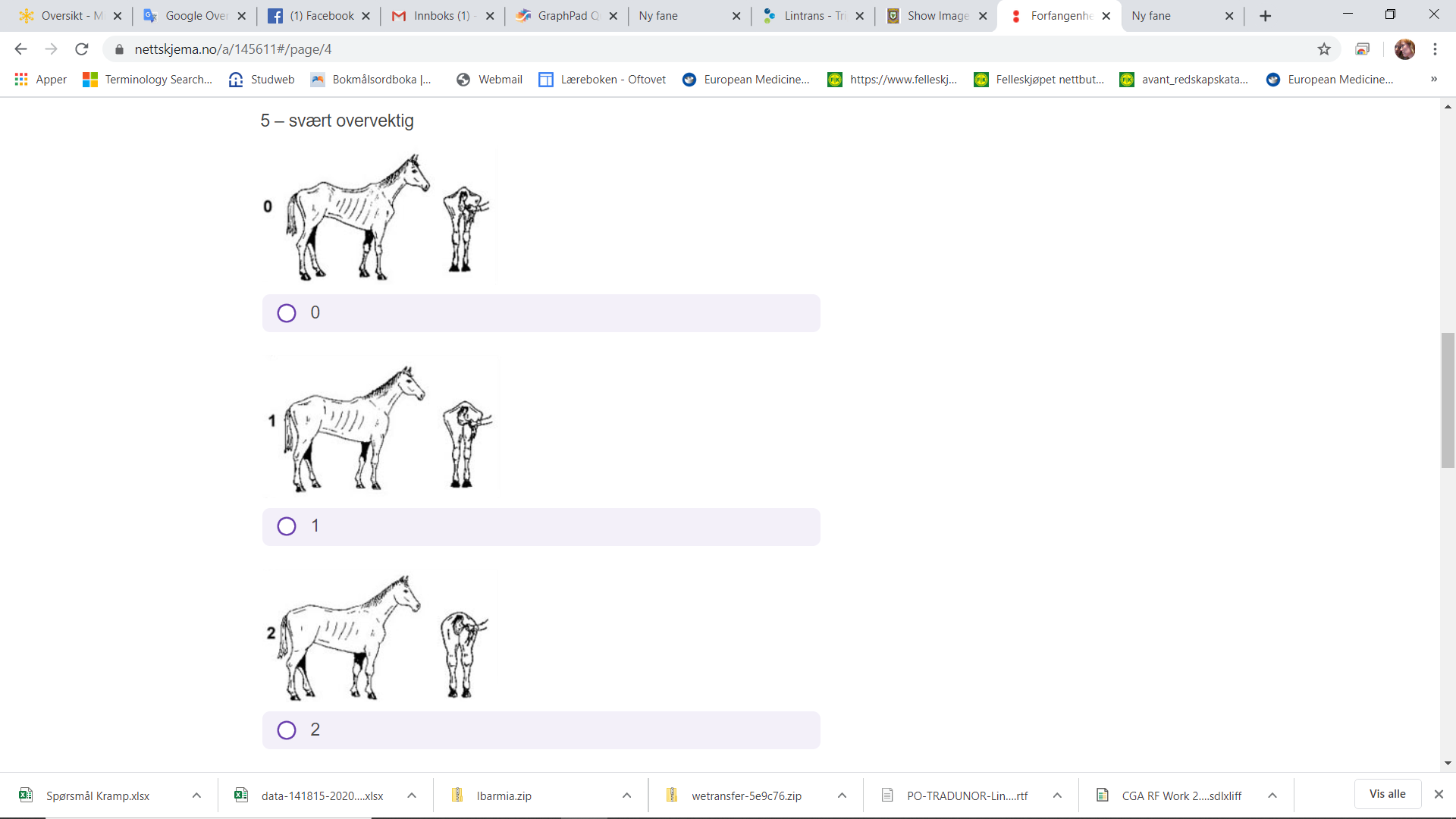

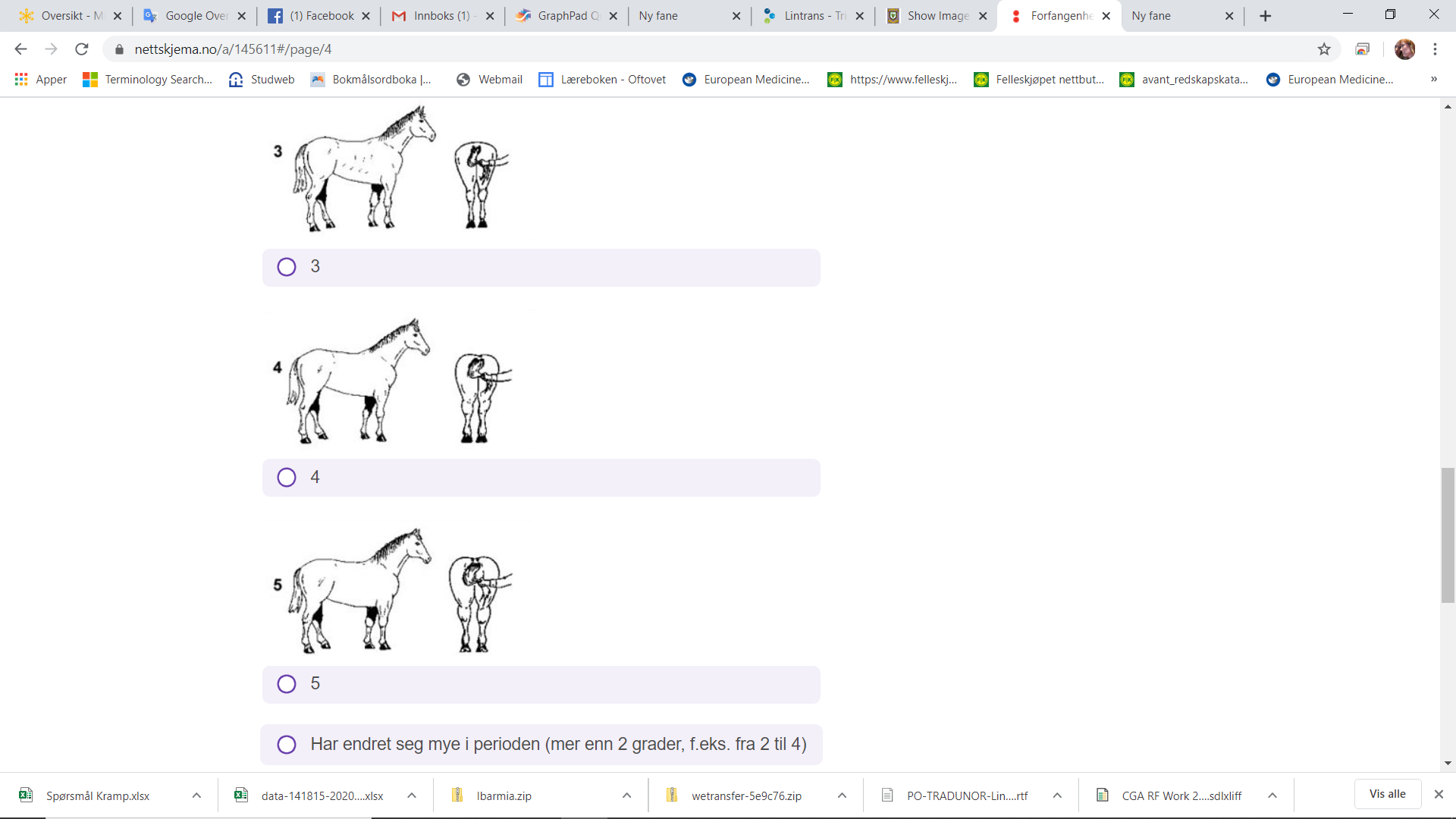


Questions regarding treatment

42. Was your horse treated for laminitis?

- Yes
- No
- Euthanized

43. Which treatment did the horse receive in the start?

- Box rest
- Soft bedding (e.g. turf)
- Styrofoam support pads
- Trimming of the toe
- NSAIDs (e.g. Flunixin, Metacam)
- Cooling (e.g. cold water or ice)

44. What was the outcome of the treatment?

- Healthy horse without relapse
- Healthy horse, but relapsed
- Did not get better, got euthanized
- Currently under treatment
- Chronic laminitis

45. Which treatment is the horse receiving for the chronic laminitis?

Choose all relevant alternatives.

- Trimming of the toe and lowering of the heel
- Corrective shoeing (heartbar or eggbar shoes, soles etc)
- Low-sugar diet
- Other corrective trimming/shoeing

46. How many recurrent episodes have your horse suffered?

- 1
- 2
- 3 or more
- Chronic laminitis
