# Supplementary material for: Prevalence and risk factors for laminitis within the Norwegian pony breed Nordlandshest/Lyngshest
Source: Acta Vet Scand. 2023 Jun 16;65:22. doi: 10.1186/s13028-023-00687-w (PMC10276406; doi:10.1186/s13028-023-00687-w)
Supplement: Supplementary file 2 — Additional file 2: Causal diagram for laminitis. [file 13028_2023_687_MOESM2_ESM.docx]

Corticosteroids
