# Supplementary material for: Prevalence and risk factors for laminitis within the Norwegian pony breed Nordlandshest/Lyngshest
Source: Acta Vet Scand. 2023 Jun 16;65:22. doi: 10.1186/s13028-023-00687-w (PMC10276406; doi:10.1186/s13028-023-00687-w)
Supplement: Supplementary file 3 — Additional file 3: Distribution across counties. The table displays the distribution across counties for all horses registered in the Norwegian breed association for Nordlandshest/lyngshestin 2019, and the NL study-population. [file 13028_2023_687_MOESM3_ESM.docx]

**Additional file 3.** Distribution across counties for horses registered in the Norwegian breed association for Nordlandshest/lyngshest (NL) in 2019 (*n* = 3022) and the NL study-population (*n* = 464).

| County affiliation | Total  Population | Study-population | Percentage included in the study. |
| --- | --- | --- | --- |
| Agder  Innlandet  Møre & Romsdal  Nordland  Oslo  Rogaland  Troms & Finnmark  Trøndelag  Vestfold & Telemark  Vestland  Viken  Total | 194  394  191  360  17  207  510  295  155  299  400  **3022** | 16  42  12  99  7  23  111  48  16  42  48  **464** | 8.2 %  10.7 %  6.3 %  27.5 %  41.2 %  11.1 %  21.8 %  16.3 %  10.3 %  14.0 %  12.0 %  **100.0 %** |
