# Supplementary material for: Prevalence and risk factors for laminitis within the Norwegian pony breed Nordlandshest/Lyngshest
Source: Acta Vet Scand. 2023 Jun 16;65:22. doi: 10.1186/s13028-023-00687-w (PMC10276406; doi:10.1186/s13028-023-00687-w)
Supplement: Supplementary file 4 — Additional file 4: Univariate analyses. The table displays the number of observations, odds ratio, 95% confidence intervalsand P-values for factors associated with outcomewith P<0.2, based on univariable testing using logistic regression. [file 13028_2023_687_MOESM4_ESM.docx]

**Additional file 4.** Number of observations (*n*), odds ratio (OR), 95% confidence intervals (CI) and *P*-values for factors associated with outcome (laminitis within the 3-year period) with *P*<0.2, based on univariable testing using logistic regression.

| Variable | *n* | OR | 95% CI of OR | *P* | *Overall P (prob>chi2)* |
| --- | --- | --- | --- | --- | --- |
| Gender  Stallion  Gelding  Mare | 71  156  236 | Reference  4.79  9.04 | 0.60-38.20  1.20-67.78 | 0.14  0.03 | 0.01 |
| Age  < 9 years  10-14 years  15-19 years  >20 years | 189  103  81  90 | Reference  4.41  4.30  3.39 | 1.62-11.97  1.51-12.26  1.17-9.84 | 0.004  0.006  0.025 | 0.01 |
| Exercise, days/week  0-3 days/week  4-7 days/week | 326  137 | 2.37  Reference | 0.97-5.82 | 0.058 | 0.04 |
| Pasture  Outfields  Infields & outfields  None | 146  305  12 | Reference  1.77  19.86 | 0.75-4.20  5.08-77.58 | 0.19  0.00 | 0.00 |
| Roughage  Hay  Vacuum-packed grass  Grass silage  Combination of roughages | 95  186  32  150 | Reference  0.44  0.65  0.45 | 0.19-1.00  0.17-2.45  0.19-1.07 | 0.05  0.53  0.07 | 0.21 |
| Quality of roughage  H1-H2  H3  H4-H5  Unknown | 76  134  65  188 | 0.48  Reference  0.45  0.47 | 0.17-1.37  0.15-1.40  0.22-1.02 | 0.17  0.17  0.06 | 0.20 |
| Analysis of roughage  Yes  No | 151  312 | 1.56  Reference | 0.79-3.07 | 0.20 | 0.20 |
| Concentrates  High energy product  Low energy product  High & low energy  None | 99  144  55  165 | 0.57  1.44  0.31  Reference | 0.20-1.64  0.68-3.04  0.09-1.85 | 0.30  0.33  0.24 | 0.13 |
| Fruits & vegetables  Yes  No | 107  356 | 0.48  Reference | 0.18-1.26 | 0.14 | 0.10 |
| Regional adiposity  No  Yes | 359  104 | Reference  3.15 | 1.59-6.22 | 0.001 | 0.00 |
| Body condition score  0-3  4-5 | 273  180 | Reference  2.52 | 1.28-4.97 | 0.01 | 0.01 |
